# Supplementary material for: Refining soil nutrient assessment: Incorporating land use boundaries for precision agriculture
Source: PLoS One. 2024 Sep 9;19(9):e0308423. doi: 10.1371/journal.pone.0308423 (PMC11383206; doi:10.1371/journal.pone.0308423)
Supplement: Attachment — Submitted filename: Reviewer Report.pdf [file pone.0308423.s001.pdf]

Review Report

Title: Refining soil nutrient assessment: incorporating land use boundaries for precision agriculture

Authors: Quan Xu and Junling He

Journal: PLOS ONE

Manuscript ID: PONE-D-24-18114

Summary of the Manuscript

The manuscript presents a study focused on refining soil nutrient assessment by incorporating land use boundaries for precision agriculture. The study utilizes soil sample data and GIS spatial interpolation analysis in Caohu Town, Xinjiang, to propose a comprehensive evaluation method for assessing soil nutrient levels at the plot level.

Detailed Review

Introduction

The introduction effectively establishes the importance of soil nutrient assessment for agricultural productivity and sustainability. The authors provide a well-structured literature review, highlighting significant advancements in the field and contextualizing their research within existing studies. The objective of the study is clearly stated, focusing on refining soil nutrient assessment by incorporating land use boundaries, which aligns with the goal of precision agriculture.

Strengths

- The topic is highly relevant given the importance of precision agriculture and optimizing soil nutrient management.
- The use of GIS spatial interpolation analysis provides a robust approach to assessing soil nutrient distribution.
- The results offer valuable insights into soil nutrient levels, which can inform better agricultural practices.

Areas for Improvement

1. Include more recent studies to provide a broader context and highlight the latest advancements in the field.
    - **Current Studies**: The introduction should include more recent studies to provide a broader context and highlight the latest advancements in the field. This not only

demonstrates the authors' awareness of the current state of research but also helps to position their study within the broader academic conversation. For example:

- **Recent Advances and Comparative Studies**: Recent studies have demonstrated significant advancements in the fields of soil nutrient assessment, particularly through the use of modern techniques such as remote sensing and machine learning. For example, Abdulraheem et al. (2023) reviewed the advancements in remote sensing for soil measurements, showcasing the potential of these technologies to enhance soil management practices. Comparative studies in other agricultural regions have provided valuable insights into the effectiveness of various soil assessment methods.

- **Example Citation**:

  Abdulraheem, M.I.; Zhang, W.; Li, S.; Moshayedi, A.J.; Farooque, A.A.; Hu, J. (2023). Advancement of Remote Sensing for Soil Measurements and Applications: A Comprehensive Review. *Sustainability, 15*(15444)

2. Provide a brief overview of the GIS spatial interpolation techniques and how plot boundary information was integrated.

- **GIS and Spatial Interpolation Techniques**

  GIS technology allows researchers to visualize and analyze spatial data, making it an invaluable tool in agricultural research. By using spatial interpolation methods such as kriging and inverse distance weighting (IDW), it is possible to create continuous surface maps that accurately represent soil nutrient levels across a study area. These methods enable researchers to account for spatial variability and provide precise nutrient assessments at the plot level

3. Clarify any technical terms to ensure accessibility for a broader audience.

- **Key Technical Terms**

  To ensure clarity, key technical terms are defined as follows: Ammonium nitrogen (AN) is a form of nitrogen readily available for plant uptake. Available phosphorus (AP) and available potassium (AK) are essential macronutrients for plant growth, while soil organic matter (SOM) is crucial for maintaining soil structure and fertility. Spatial interpolation refers to the process of estimating unknown values at certain locations based on known values from surrounding locations. GIS technology facilitates the integration and analysis of spatial data.

4. Strengthen the rationale for the study

- **Rationale and Impact of the Study**

  Effective soil nutrient management is critical for optimizing crop yields and ensuring sustainable agricultural practices. However, traditional soil assessment

methods often lack the precision needed for targeted fertilization. This study aims to address nutrient management challenges by incorporating plot boundary information into soil nutrient assessments. By providing farmers with more accurate data, the study aims to enhance fertilization strategies, improve crop production efficiency, and contribute to sustainable agricultural practices.

5. Highlight the novelty and contributions of the study

- **Novelty and Contributions**

  This study distinguishes itself by integrating plot boundary information into the spatial interpolation of soil nutrient levels. This novel approach enhances the precision of nutrient assessments, providing a more refined basis for scientific fertilization practices in Caohu Town, Xinjiang. The findings are expected to offer valuable insights for farmers, policymakers, and researchers, advancing the field of precision agriculture and soil nutrient management.

Methodology

The methodology is well-detailed, describing the study area, soil sampling and analysis, spatial interpolation methods, and the comprehensive evaluation method. The authors effectively use GIS technology to integrate soil nutrient data with spatial interpolation results, enhancing the precision of soil nutrient evaluation.

Strengths

- Detailed description of the study area sets a clear context for the research.
- Standard methods for soil sampling and laboratory analysis ensure reliable data collection.
- The use of kriging, IDW, and GIS technology is appropriate for studying spatial distribution patterns of soil nutrients.

Areas for Improvement

- Provide more specific details on the spatial interpolation techniques and parameters used to ensure reproducibility.
- Include a section on the validation of the spatial interpolation methods to demonstrate their accuracy and reliability.
- Expand on the practical applications of the findings, including specific recommendations for farmers and agricultural managers.

Results

The results section presents a clear and comprehensive analysis of soil nutrient levels and their spatial distribution. The descriptive statistical analysis provides an understanding of overall soil nutrient levels and their variation within the study area.

Strengths

- The descriptive statistical analysis provides a clear understanding of the overall soil nutrient levels and their variation within the study area.
- The spatial distribution maps and analysis effectively illustrate the nutrient distribution patterns and highlight areas with different nutrient levels.
- The comprehensive evaluation categorizes soil nutrient levels into different grades, offering practical implications for soil management and fertilization practices.

Areas for Improvement

- Ensure that the figures and maps are clear and well-labeled to enhance readability and understanding of the spatial distribution patterns.
- Provide more detailed explanations of the spatial distribution patterns and their implications for soil management and crop production.
- Expand the discussion on how the results can inform specific fertilization strategies and soil management practices in the study area.

Discussion

The discussion highlights the positive results of the study and suggests areas for further exploration. The proposed comprehensive evaluation method is beneficial for farmers to understand soil nutrient levels and implement scientific fertilization management.

Strengths

- The discussion emphasizes the practical applications of the findings for precision agriculture.
- It provides a clear summary of the study's contributions and the importance of incorporating plot boundary information into soil nutrient assessments.

Areas for Improvement

- Explore the use of objective weighting methods for assigning values to soil nutrient indicators to reduce subjective biases.
- Include a section on the validation of spatial interpolation results using training and validation sets to improve the accuracy of the method.
- Provide more detailed discussions on the practical implications of the findings for farmers and agricultural managers.

Conclusion

The conclusion effectively summarizes the key findings of the study and provides recommendations for future research and practical applications. The authors emphasize the importance of implementing the findings to achieve better soil management, enhance crop production, and contribute to sustainable agriculture.

Strengths

- The conclusion provides a clear summary of the study's key findings and their practical implications.
- It highlights the importance of refining and enhancing the comprehensive evaluation method for broader applicability and accuracy.

Areas for Improvement

- Suggest specific areas for future research to further refine and enhance the comprehensive evaluation method.
- Emphasize the practical applications of the findings, including specific recommendations for farmers on fertilization strategies and soil management practices.

Final Recommendation

Based on the detailed review, I find that the manuscript presents significant contributions to the field of soil nutrient assessment and precision agriculture. The methodology is robust, and the results offer valuable insights for improving soil management and crop production. However, the manuscript would benefit from addressing the areas for improvement mentioned above.

Recommendation: Minor Revisions

Specific Suggestions for Authors:

1. Expand the literature review to include more recent studies.
2. Provide more specific details on the spatial interpolation techniques and parameters used.
3. Include a section on the validation of the spatial interpolation methods.
4. Ensure that figures and maps are clear and well-labeled.
5. Provide more detailed explanations of the spatial distribution patterns and their practical implications.
6. Explore the use of objective weighting methods for assigning values to soil nutrient indicators.
7. Expand on the practical implications of the findings for farmers and agricultural managers.

8. Suggest specific areas for future research.

I believe addressing these suggestions will enhance the manuscript's clarity, reliability, and practical relevance.

Sincerely,

Bright Fafali Dogbey
