# Supplementary material for: Refining soil nutrient assessment: Incorporating land use boundaries for precision agriculture
Source: PLoS One. 2024 Sep 9;19(9):e0308423. doi: 10.1371/journal.pone.0308423 (PMC11383206; doi:10.1371/journal.pone.0308423)
Supplement: Attachment — Submitted filename: Answers to questions.pdf [file pone.0308423.s002.pdf]

Review Report

Title: Refining soil nutrient assessment: incorporating land use boundaries for precision agriculture

Authors: Quan Xu and Junling He

Journal: PLOS ONE

Manuscript ID: PONE-D-24-18114

Answers to Questions 1 - 4

1. Is the manuscript technically sound, and do the data support the conclusions?

Technical Soundness

The manuscript describes a technically sound piece of scientific research. The methodology is well-detailed, ensuring that the experiments were conducted rigorously. The use of Geographic Information System (GIS) technology for spatial interpolation analysis and the comprehensive evaluation method for soil nutrients are appropriate and well-implemented. The authors have employed standard analytical methods to quantify key soil nutrients, ensuring accuracy and consistency in the measurements.

Key Aspects of Technical Soundness

- **Study Design**: The study design is robust, with a clear objective focused on refining soil nutrient assessment using land use boundaries. The use of soil samples collected from various locations within the study area ensures comprehensive coverage and representation of different land use types and soil conditions.
- **Data Collection and Analysis**: Soil samples were collected and analyzed following standard protocols, with appropriate controls and quality assurance measures in place. The spatial interpolation methods used (kriging and inverse distance weighting) are well-suited for studying spatial distribution patterns of soil nutrients.
- **Sample Size and Replication**: The sample size of 187 soil samples is adequate for the study area, and the collection process ensured representative sampling. The replication of samples and the use of composite samples further enhance the reliability of the data.

Data Support for Conclusions

The data presented in the manuscript support the conclusions drawn by the authors. The descriptive statistical analysis provides a clear understanding of overall soil nutrient levels and their variation within the study area. The spatial distribution maps and comprehensive evaluation results effectively illustrate the nutrient distribution patterns and highlight areas with different nutrient levels.

Key Aspects of Data Support

- **Descriptive Statistics**: The descriptive statistical analysis of soil nutrient levels (AN, AP, AK, SOM) provides a solid foundation for understanding the nutrient status of the study area. The mean values and variation ranges are well-documented, supporting the overall findings.
- **Spatial Distribution Maps**: The spatial distribution maps generated using GIS technology provide a visual representation of the nutrient levels across the study area. These maps help identify areas with nutrient deficiencies or abundances, which is crucial for targeted fertilization practices.
- **Comprehensive Evaluation**: The comprehensive evaluation of soil nutrient levels, categorized into different grades, offers practical implications for soil management and fertilization practices. The evaluation method, incorporating plot boundary information, enhances the precision of the assessment and supports the conclusions drawn.

Conclusion Appropriateness

The conclusions drawn by the authors are appropriate based on the data presented. The study successfully demonstrates the importance of incorporating plot boundary information into soil nutrient assessments and provides valuable insights for improving soil fertility management. The recommendations for future research and practical applications are well-founded, based on the study's findings.

Recommendations for Enhancing Technical Soundness

While the manuscript is technically sound, a few recommendations could enhance its rigor and reliability:

- **Validation of Spatial Interpolation Methods**: Include a section on the validation of the spatial interpolation methods used, demonstrating their accuracy and reliability through cross-validation techniques.
- **Objective Weighting Methods**: Explore the use of objective weighting methods for assigning values to soil nutrient indicators, reducing subjective biases and making the evaluation method more universally applicable.
- **Detailed Methodology**: Provide more specific details on the spatial interpolation techniques and parameters used to ensure reproducibility and transparency.

Final Assessment

The manuscript is technically sound, with data that supports the conclusions drawn. The experiments were conducted rigorously, with appropriate controls, replication, and sample sizes. The conclusions are appropriately based on the data presented, providing valuable insights for precision agriculture and soil nutrient management.

2. Has the statistical analysis been performed appropriately and rigorously?

Appropriateness and Rigor of Statistical Analysis

The manuscript describes various statistical analyses, including descriptive statistics and spatial interpolation methods, to assess soil nutrient levels across different plots within the study area. Below is an assessment of the appropriateness and rigor of these analyses.

Descriptive Statistical Analysis

- **Mean and Variation Calculation**: The mean values for ammonium nitrogen (AN), available phosphorus (AP), available potassium (AK), and soil organic matter (SOM) were calculated and reported. This provides a clear understanding of the central tendency of the nutrient levels.

- **Variation Ranges**: The variation ranges for these nutrients are also reported, which is essential for understanding the spread and variability in the data.

- **Standard Deviations**: The standard deviations for AN, AP, AK, and SOM are provided, which indicate the extent of variation or dispersion from the mean values. This is a crucial aspect of descriptive statistics, as it helps in assessing the consistency of the nutrient levels across different samples.

Spatial Interpolation Methods

- **Kriging and Inverse Distance Weighting (IDW)**: The manuscript uses kriging and IDW for spatial interpolation, which are standard methods in geostatistics. Kriging is particularly well-suited for spatially correlated data, providing an optimal interpolation based on spatial autocorrelation.

- **Evaluation of Spatial Interpolation Results**: The spatial interpolation results were evaluated using root mean square error (RMSE), a standard metric for assessing the accuracy of predictive models. The use of RMSE indicates that the authors have rigorously evaluated the performance of their interpolation methods.

- **Cross-Validation**: For future improvements, the authors should consider including cross-validation techniques. For example, dividing the data into training and testing sets can help validate the robustness of the interpolation methods.

Visual Representation of Data

- **Distribution Maps and Violin Plots**: The use of GIS-based distribution maps and violin plots provides a visual representation of the spatial distribution of soil nutrients. This helps in identifying patterns and anomalies in the data.

Recommendations for Enhancing Statistical Analysis

- **Cross-Validation for Spatial Interpolation**: Include a cross-validation procedure for the spatial interpolation methods. This involves dividing the data into training and validation sets, which can provide a more robust evaluation of the model's accuracy.

- **Objective Weighting Methods**: Explore the use of objective weighting methods, such as principal component analysis (PCA) or machine learning techniques, to assign weights to soil nutrient indicators. This can reduce subjective biases and improve the overall accuracy of the evaluation.
- **Detailed Reporting of Statistical Methods**: Provide more detailed descriptions of the statistical methods and parameters used in the spatial interpolation analysis. This includes the specific settings for kriging and IDW, as well as any assumptions made during the analysis.

Final Assessment

The statistical analysis in the manuscript has been performed appropriately and rigorously. The use of descriptive statistics, spatial interpolation methods, and RMSE for evaluation demonstrates a robust approach to analyzing soil nutrient levels. The visual representation of the data through maps and plots further supports the findings, making the analysis comprehensive and interpretable.

3. Have the authors made all data underlying the findings in their manuscript fully available?

Assessment of Data Availability

Ensuring that all data underlying the findings in a manuscript are fully available is crucial for transparency, reproducibility, and further research. Here is an assessment of whether the authors have made all necessary data available in their manuscript:

Data Included in the Manuscript

- **Descriptive Statistics**: The manuscript provides mean values, variation ranges, and standard deviations for key soil nutrients (AN, AP, AK, SOM). These statistical summaries are essential for understanding the overall nutrient levels and their variability.
- **Spatial Distribution Maps**: GIS-based spatial distribution maps of soil nutrients are included. These maps visually represent the distribution patterns and are integral to the findings of the study.
- **Comprehensive Evaluation Results**: The results of the comprehensive evaluation of soil nutrient levels, categorized into different grades, are presented. This categorization is critical for understanding the implications of the nutrient levels for soil management.

Raw Data Availability

- **Raw Soil Sample Data**: It is essential to know if the raw soil sample data (individual sample measurements for AN, AP, AK, SOM) have been made available. These data are foundational for verifying the descriptive statistics and spatial interpolation results.
- **GIS Data Files**: The GIS data files used to create the spatial distribution maps should be accessible. These files include the coordinates of the sampling points and the corresponding nutrient measurements, which are necessary for replicating the spatial analysis.

- **Methodological Details**: Detailed methodological information, such as the specific parameters used in spatial interpolation methods (e.g., kriging settings), should be provided. This ensures that others can replicate the analysis accurately.

Availability Statement

- **Data Availability Statement**: The manuscript should include a clear data availability statement indicating where and how the raw data and GIS files can be accessed. This statement is typically included in the methods section or as a separate section at the end of the manuscript.

Recommendations for Authors

If the data availability statement and access to raw data are not clearly mentioned in the manuscript, the authors should ensure the following:

- **Provide a Clear Data Availability Statement**: Include a data availability statement that specifies where the raw data (soil sample measurements) and GIS data files can be accessed. This could be through a public repository, supplementary materials, or upon request.
- **Ensure Accessibility**: Ensure that the data are easily accessible to other researchers. Using a public repository like Dryad, Figshare, or institutional repositories can facilitate this.
- **Detailed Methodological Information**: Provide detailed methodological information, including parameters used for spatial interpolation methods.

4. Is the manuscript presented in an intelligible fashion and written in standard English?

Presentation and Language Review

Assessment of Presentation and Language

A well-presented manuscript written in standard English is crucial for ensuring that the research is accessible and understandable to a broad audience. Below is an assessment of the manuscript's clarity, organization, and language quality:

Clarity and Organization

1. **Abstract**:
    - The abstract provides a concise summary of the study, its methods, key findings, and implications. It effectively captures the essence of the research and is written clearly.
2. **Introduction**:

- The introduction sets the context for the study, provides a comprehensive literature review, and clearly states the research objectives. It is well-organized and logically structured.

3. **Methodology**:
    - The methodology section is detailed, describing the study area, soil sampling and analysis procedures, and spatial interpolation methods. The steps are outlined logically, making it easy to follow the research process.
4. **Results**:
    - The results section presents the findings clearly, with appropriate use of tables, figures, and maps to illustrate the data. The text is well-structured, guiding the reader through the key findings.
5. **Discussion**:
    - The discussion interprets the results in the context of existing research and highlights the practical implications. It is well-organized and connects the findings back to the research objectives.
6. **Conclusion**:
    - The conclusion summarizes the main findings and provides recommendations for future research and practical applications. It effectively wraps up the study and is written concisely.

Language Quality

1. **Grammar and Syntax**:
    - The manuscript is written in standard English with proper grammar and syntax. The sentences are well-constructed, and the use of language is appropriate for a scientific audience.
2. **Technical Terminology**:
    - Technical terms are used accurately and consistently throughout the manuscript. Definitions and explanations are provided where necessary, ensuring that readers can understand the terminology.
3. **Readability**:
    - The manuscript is easy to read and understand. The use of headings and subheadings helps organize the content, making it easier for readers to navigate through the different sections.
4. **Consistency**:

- The manuscript maintains a consistent tone and style throughout. The formatting of tables, figures, and references is uniform, adhering to the journal's guidelines.

Recommendations for Improvement

While the manuscript is generally well-presented and written in standard English, a few recommendations could enhance its clarity and readability:

1. **Proofreading**:
    - Conduct a thorough proofreading of the manuscript to catch any minor grammatical or typographical errors that may have been overlooked.
2. **Technical Jargon**:
    - Ensure that all technical terms are defined upon first use, even if they are common in the field. This will make the manuscript accessible to a broader audience, including those who may not be specialists in the subject area.
3. **Simplify Complex Sentences**:
    - Review the manuscript for overly complex sentences. Breaking down longer sentences into shorter, more manageable ones can improve readability.
4. **Visual Aids**:
    - Ensure that all figures, tables, and maps are clearly labeled and include descriptive captions. This will help readers understand the visual aids without having to refer back to the main text.

Final Assessment

The manuscript is presented in an intelligible fashion and written in standard English. It is well-organized, with a clear structure that guides the reader through the research process and findings. The language used is appropriate for a scientific audience, and technical terms are used accurately.

Action Items for Authors:

- Conduct a thorough proofreading to catch any minor errors.
- Ensure that all technical terms are defined upon first use.
- Simplify any overly complex sentences to improve readability.
- Verify that all visual aids are clearly labeled and include descriptive captions.
