# Supplementary material for: Refining soil nutrient assessment: Incorporating land use boundaries for precision agriculture
Source: PLoS One. 2024 Sep 9;19(9):e0308423. doi: 10.1371/journal.pone.0308423 (PMC11383206; doi:10.1371/journal.pone.0308423)
Supplement: Attachment — Submitted filename: Response to Reviewers.docx [file pone.0308423.s003.docx]

I would like to express my heartfelt respect and gratitude to the expert for your meticulous and professional revision in your busy schedule, which makes the article more rigorous and further improved. Thanks to the editor and experts for the opportunity to revise. If there is any problem, please feel free to contact me at any time. I am very willing to make positive changes.

**Reviewer #1**

Thank you for inviting me to review the paper entitled “Refining soil nutrient assessment: incorporating land use boundaries for precision agriculture”. The topic of the article is interesting. I recommend reconsideration of the paper after addressing the following modifications:
1.What is the novelty of this manuscript? It should be clearly mentioned in the last paragraph of the introduction.

**Response 1:** The expert advice has been fully adopted. The structure of the introduction is adjusted, and the innovation of this study is mentioned in the last paragraph. Please see the introduction for details.

2.The introduction needed to be expanded and the literature review needed to be updated with up-to-date references about contamination due to land use and its effects on soil nutrients, e.g.: [https://doi.org/10.1007/978-3-031-55665-4_2](https://doi.org/10.1007/978-3-031-55665-4_2)

**Response 2:** The introduction has been supplemented with expert comments, the effects of land use on soil nutrients have been updated, and the literature has been cited.

3.Please, replace all the figures with high-resolution ones.

**Response 3:** The expert's advice has been fully adopted. All image resolutions have been modified and will be packaged and uploaded as attachments.

4.Figs.1 & 4 & 5 & 6 have not any spatial coordinates. Please, provide them.

**Response 4:** Coordinates have been added to the picture based on expert advice.

5.The font of legends is very small. Please, give a suitable font size for legends.

**Response 5:** We have modified the legend in the full text according to the advice of experts.

6.You should add the limitation of your study at the end of the paper before the conclusion section.

**Response 6:** The expert advice has been fully adopted. In the discussion section, the limitations of this study and the focus of future research are described.

7.What is your recommendation for future studies to enhance the applied methodology? You can discuss how to relate your research findings with sustainable development goals (SDGs) to achieve more environmental, economic, and social benefits. References to cite:
[https://doi.org/10.1016/j.ejrh.2024.101703](https://doi.org/10.1016/j.ejrh.2024.101703)
[https://doi.org/10.1016/j.gsd.2024.101087](https://doi.org/10.1016/j.gsd.2024.101087)
[https://doi.org/10.1007/978-981-99-4101-8_27](https://doi.org/10.1007/978-981-99-4101-8_27)
[https://doi.org/](https://doi.org/10.1007/978-981-99-1381-7_6)[10.1007/978-981-99-1381-7_6](https://doi.org/10.1007/978-981-99-1381-7_6)

**Response 7:** We have supplemented the discussion with references based on expert advice. Research findings are linked to the Sustainable Development Goals and priorities for future research are discussed. Please see the discussion section for details.

**Reviewer #2**

Review report uploaded as an attachment since it exceeds 20,000 characters. All 4 questions have been answered and explained thoroughly. Additional comments for the author has also been added. The file is named answers to questions.

Summary of the Manuscript

The manuscript presents a study focused on refining soil nutrient assessment by incorporating land use boundaries for precision agriculture. The study utilizes soil sample data and GIS spatial interpolation analysis in Caohu Town, Xinjiang, to propose a comprehensive evaluation method for assessing soil nutrient levels at the plot level.

Detailed Review

**Introduction**

The introduction effectively establishes the importance of soil nutrient assessment for agricultural productivity and sustainability. The authors provide a well-structured literature review, highlighting significant advancements in the field and contextualizing their research within existing studies. The objective of the study is clearly stated, focusing on refining soil nutrient assessment by incorporating land use boundaries, which aligns with the goal of precision agriculture.

Strengths

•The topic is highly relevant given the importance of precision agriculture and optimizing soil nutrient management.

•The use of GIS spatial interpolation analysis provides a robust approach to assessing soil nutrient distribution.

•The results offer valuable insights into soil nutrient levels, which can inform better agricultural practices.

Areas for Improvement

1. Include more recent studies to provide a broader context and highlight the latest advancements in the field.

•Current Studies: The introduction should include more recent studies to provide a broader context and highlight the latest advancements in the field. This not only demonstrates the authors' awareness of the current state of research but also helps to position their study within the broader academic conversation. For example:

•Recent Advances and Comparative Studies: Recent studies have demonstrated significant advancements in the fields of soil nutrient assessment, particularly through the use of modern techniques such as remote sensing and machine learning. For example, Abdulraheem et al. (2023) reviewed the advancements in remote sensing for soil measurements, showcasing the potential of these technologies to enhance soil management practices. Comparative studies in other agricultural regions have provided valuable insights into the effectiveness of various soil assessment methods.

•Example Citation:

Abdulraheem, M.I.; Zhang, W.; Li, S.; Moshayedi, A.J.; Farooque, A.A.; Hu, J. (2023). Advancement of Remote Sensing for Soil Measurements and Applications: A Comprehensive Review. Sustainability, 15(15444)

**Response :** We have taken the expert's advice in full. In the introduction part, the latest research results are added and the literature is cited. Please see the introduction for details.

2. Provide a brief overview of the GIS spatial interpolation techniques and how plot boundary information was integrated.

• GIS and Spatial Interpolation Techniques

GIS technology allows researchers to visualize and analyze spatial data, making it an invaluable tool in agricultural research. By using spatial interpolation methods such as kriging and inverse distance weighting (IDW), it is possible to create continuous surface maps that accurately representsoil nutrient levels across a study area. These methods enable researchers to account for spatial variability and provide precise nutrient assessments at the plot level.

**Response :** According to the suggestions of expert, we have briefly introduced GIS spatial interpolation technology and integrated plot boundary information technology in the introduction part, and used the examples given by expert for reference.

3.Clarify any technical terms to ensure accessibility for a broader audience.

• Key Technical Terms

To ensure clarity, key technical terms are defined as follows: Ammonium nitrogen (AN) is a form of nitrogen readily available for plant uptake. Available phosphorus (AP) and available potassium (AK) are essential macronutrients for plant growth, while soil organic matter (SOM) is crucial for maintaining soil structure and fertility. Spatial interpolation refers to the process of estimating unknown values at certain locations based on known values from surrounding locations. GIS technology facilitates the integration and analysis of spatial data.

**Response :** Many thanks to the expert for such detailed suggestions. We have supplemented the key technical terms in the last paragraph of the introduction.

4.Strengthen the rationale for the study

•Rationale and Impact of the Study

Effective soil nutrient management is critical for optimizing crop yields and ensuring sustainable agricultural practices. However, traditional soil assessment methods often lack the precision needed for targeted fertilization. This study aims to address nutrient management challenges by incorporating plot boundary information into soil nutrient assessments. By providing farmers with more accurate data, the study aims to enhance fertilization strategies, improve crop production efficiency, and contribute to sustainable agricultural practices.

**Response :** We have fully taken the expert's advice and supplemented the research rationale in the introduction.

5.Highlight the novelty and contributions of the study

•Novelty and Contributions

This study distinguishes itself by integrating plot boundary information into the spatial interpolation of soil nutrient levels. This novel approach enhances the precision of nutrient assessments, providing a more refined basis for scientific fertilization practices in Caohu Town, Xinjiang. The findings are expected to offer valuable insights for farmers, policymakers, and researchers, advancing the field of precision agriculture and soil nutrient management.

**Response :** We have taken the expert's advice in full. The innovation of this research is further emphasized in the introduction.

**Methodology**

The methodology is well-detailed, describing the study area, soil sampling and analysis, spatial interpolation methods, and the comprehensive evaluation method. The authors effectively use GIS technology to integrate soil nutrient data with spatial interpolation results, enhancing the precision of soil nutrient evaluation.

Strengths

• Detailed description of the study area sets a clear context for the research.

• Standard methods for soil sampling and laboratory analysis ensure reliable data collection.

•The use of kriging, IDW, and GIS technology is appropriate for studying spatial distribution patterns of soil nutrients.

Areas for Improvement

•Provide more specific details on the spatial interpolation techniques and parameters used to ensure reproducibility.

**Response :** According to the expert opinion, the parameters of spatial interpolation are supplemented in the method.

•Include a section on the validation of the spatial interpolation methods to demonstrate their accuracy and reliability.

**Response :** In order to better verify the accuracy of the model, 80% of the data set is used as the training set, and the remaining 20% is used as the training set. We have explained this in more detail in the Methods section, based on expert advice.

•Expand on the practical applications of the findings, including specific recommendations for farmers and agricultural managers.

**Response :** The expert advice has been fully adopted. Based on the evaluation results of soil nutrients, we put forward corresponding reasonable suggestions for farmers and agricultural managers to further improve soil nutrients. Please see the "Comprehensive evaluation of soil nutrients" section for details.

**Results**

The results section presents a clear and comprehensive analysis of soil nutrient levels and their spatial distribution. The descriptive statistical analysis provides an understanding of overall soil nutrient levels and their variation within the study area.

Strengths

•The descriptive statistical analysis provides a clear understanding of the overall soil nutrient levels and their variation within the study area.

•The spatial distribution maps and analysis effectively illustrate the nutrient distribution patterns and highlight areas with different nutrient levels.

•The comprehensive evaluation categorizes soil nutrient levels into different grades, offering practical implications for soil management and fertilization practices.

Areas for Improvement

•Ensure that the figures and maps are clear and well-labeled to enhance readability and understanding of the spatial distribution patterns.

**Response :** We have fully adopted the expert's advice. We have modified and improved all the pictures in the article, and modified the text and legend in the map to make it clearer. And improved the resolution of all images. All images will be uploaded together as attachments.

•Provide more detailed explanations of the spatial distribution patterns and their implications for soil management and crop production.

**Response :** We have made further supplementary explanations on the spatial distribution patterns of different soil nutrients and their effects on agricultural production management based on expert advice. Please see the results section for details.

•Expand the discussion on how the results can inform specific fertilization strategies and soil management practices in the study area.

**Response :** We have taken the expert's advice in full. In the results chapter, we discuss how farmers can specifically apply fertilizer and manage soil based on our findings.

**Discussion**

The discussion highlights the positive results of the study and suggests areas for further exploration. The proposed comprehensive evaluation method is beneficial for farmers to understand soil nutrient levels and implement scientific fertilization management.

Strengths

•The discussion emphasizes the practical applications of the findings for precision agriculture.

•It provides a clear summary of the study's contributions and the importance of incorporating plot boundary information into soil nutrient assessments.

Areas for Improvement

•Explore the use of objective weighting methods for assigning values to soil nutrient indicators to reduce subjective biases.

**Response :** According to the opinions of expert, we have made it clear in the discussion section that exploring the use of objective weight method to assign values to indicators is one of our future research priorities.

•Include a section on the validation of spatial interpolation results using training and validation sets to improve the accuracy of the method.

**Response :** Thanks for the expert advice. We divided the data set into a training set and a validation set in the form of 8:2 to evaluate the accuracy of spatial interpolation. We have made further supplementary explanations in the research methods.

•Provide more detailed discussions on the practical implications of the findings for farmers and agricultural managers.

**Response :** We have taken the expert's advice in full. The beneficial effects of the findings on farmers and agricultural managers are explained in the discussion section.

**Conclusion**

The conclusion effectively summarizes the key findings of the study and provides recommendations for future research and practical applications. The authors emphasize the importance of implementing the findings to achieve better soil management, enhance crop production, and contribute to sustainable agriculture.

Strengths

•The conclusion provides a clear summary of the study's key findings and their practical implications.

•It highlights the importance of refining and enhancing the comprehensive evaluation method for broader applicability and accuracy.

Areas for Improvement

•Suggest specific areas for future research to further refine and enhance the comprehensive evaluation method.

**Response :** We have taken the advice of the expert and made a detailed description of our future research priorities.

•Emphasize the practical applications of the findings, including specific recommendations for farmers on fertilization strategies and soil management practices.

**Response :** Based on the expert opinion, we have further emphasized the innovation of the research method and the practical application of the research results in the conclusion.

Final Recommendation

Based on the detailed review, I find that the manuscript presents significant contributions to the field of soil nutrient assessment and precision agriculture. The methodology is robust, and the results offer valuable insights for improving soil management and crop production. However, the manuscript would benefit from addressing the areas for improvement mentioned above.

Recommendation: Minor Revisions

Specific Suggestions for Authors:

1. Expand the literature review to include more recent studies.

**Response1 :** We have expanded the literature review in the introduction based on expert input, citing more recent studies.

2. Provide more specific details on the spatial interpolation techniques and parameters used.

**Response2 :** The recommendations of the expert have been fully adopted, and more specific details on spatial interpolation techniques and parameters are provided in the Methods chapter.

3.Include a section on the validation of the spatial interpolation methods.

**Response3 :** In order to better evaluate the accuracy of spatial interpolation, 80% of the data set is set as the training set, and 20% is set as the training set. According to the advice of the expert, further supplementary explanations have been made in the article.

4.Ensure that figures and maps are clear and well-labeled.

**Response4 :** We have taken the expert's advice in full. The picture in the whole article has been modified.

5.Provide more detailed explanations of the spatial distribution patterns and their practical implications.

**Response5 :** We have made a more detailed explanation of the spatial distribution in the results section based on the opinions of expert.

6.Explore the use of objective weighting methods for assigning values to soil nutrient indicators.

**Response6 :** We have adopted the advice of expert and mentioned in the discussion section that exploring the use of objective weighting methods to assign values to soil nutrient indicators is one of our future priorities.

7.Expand on the practical implications of the findings for farmers and agricultural managers.

**Response7 :** The expert advice has been fully adopted. The practical implications of the research results for farmers and agricultural managers are further explained in several chapters.

8.Suggest specific areas for future research.

**Response8 :** Based on expert advice, we have further identified specific areas for future research in the discussion section.

I believe addressing these suggestions will enhance the manuscript's clarity, reliability, and practical relevance.

Sincerely,

Bright Fafali Dogbey

**Reviewer #3**

1. The introduction section provides a good overview of the importance of soil nutrient assessment, but it lacks a comprehensive review of the existing literature on similar methodologies. Could you expand on the existing methods for soil nutrient evaluation at the plot level and highlight the novelty or advantages of your proposed approach compared to those methods?
   **Response 1:** We have taken the expert's advice in full. We supplemented the latest research advances in soil nutrient assessment and cited relevant literature. At the same time, at the end of the introduction, we made a more detailed introduction to the innovation of the research method, that is, compared with the previous methods, we further improved the assessment accuracy by introducing the plot boundary information. Please see the introduction for detailed changes.

2. In the sample collection and processing section, you mention that both external and internal quality control methods were employed to ensure the reliability of the soil sample analysis. Could you provide more details on the specific external and internal quality control measures taken, as this would help assess the robustness of your data?
**Response 2:** We have supplemented our external and internal quality control laws with expert advice. Please see the “Sample Collection and Processing” section for details.

3. The spatial interpolation method used in this study is Kriging. While Kriging is a widely used technique, there are various other interpolation methods available (e.g., Inverse Distance Weighting, Radial Basis Functions, etc.). Could you justify the selection of Kriging over other methods and discuss the potential advantages or limitations of using Kriging for this particular study?
**Response 3:** Thanks to the expert for this significant advice. This will be of great help to our research work. In order to explore the impact of the introduction of plot information on the accuracy of soil nutrient evaluation results, the most commonly used Kriging interpolation method was selected according to previous research results. The advantage of this is that it can clearly reflect whether our research methods can improve the accuracy of evaluation. Therefore, we do not try different spatial interpolation methods. We further clarified in the discussion section that exploring different spatial interpolation methods is one of our future priorities. Thanks to the expert for this valuable advice, which inspired us a lot.

4. In the comprehensive evaluation of soil nutrients section, you mention that the weights for AN, AP, AK, and SOM were set based on standards and related research results. However, the rationale behind the specific weight assignment is not explained. Could you provide more details on how these weights were determined and discuss the potential impact of different weight assignments on the final evaluation results?

**Response 4:** Thanks to the expert for this valuable advice, which is of great help to the article improvement. There is no doubt that different elements have different effects on soil nutrients. Therefore, it is necessary to assign different indicators to different elements to reflect their contribution to soil nutrients. Our research is mainly carried out with reference to Land Quality Evaluation Standards (DZ/T0925-2016), which is formed after repeatedly consulting many opinions and suggestions and is the most suitable standard for evaluating soil nutrients in China (Geochemistry). A lot of research work has been carried out according to this standard. Therefore, we further add in the discussion part that in future work, we will explore the use of objective weighting methods to assign values in different regions, so as to make the evaluation method more generally applicable

5. The research focuses on evaluating soil nutrient levels within a specific study area. Could you discuss the potential applicability and transferability of your proposed method to other regions or crops, considering potential variations in soil conditions, climate, and agricultural practices?
**Response 5:** We have fully adopted the expert's advice. The potential applicability of this method in other areas is further explained in the discussion section. At the same time, in the future, we will use the model to further carry out work in different regions, and constantly enrich and improve the model.

6. The study primarily focuses on the spatial distribution and evaluation of soil nutrients. However, it does not address the temporal aspect of soil nutrient dynamics. Could you discuss the potential implications of considering temporal variations in soil nutrient levels and how your proposed method could be adapted or extended to account for such variations?
**Response 6:** We have fully adopted the expert's advice. The temporal dynamics of soil nutrients in our proposed model were supplemented. In the future, we will build time-series data, and then propose a new soil nutrient time-series evaluation model to better reflect the characteristics of soil nutrient changes in time.

7. The comprehensive evaluation of soil nutrients is based on a weighted overlay method. While this method provides a single overall score, it may not capture the potential interactions or trade-offs between different nutrient components. Could you discuss the limitations of this approach and explore alternative methods that could provide a more nuanced understanding of soil nutrient dynamics?
**Response 7:** We have discussed the limitations of our current approach based on expert advice. In the future, we will continue to do a lot of work to continuously modify and improve our model to make it more accurate and usable. We will evaluate soil nutrients more accurately from the two dimensions of time and space.

8. The study provides valuable insights into soil nutrient levels and their spatial distribution within the study area. However, it does not address the potential causes or drivers of the observed patterns. Could you discuss potential factors (e.g., land use, management practices, environmental conditions) that may influence the spatial distribution of soil nutrients and how these factors could be incorporated into future research?

**Response 8:** We have fully adopted the expert's advice. We mentioned in the discussion section that we will conduct research in more regions in the future. Different land use methods and different environmental conditions will be taken as variables, and comparative experiments will be carried out to deeply explore their effects on soil nutrients.

Thanks again to the experts for your efforts!

**#PLOS ONE's style requirements**

When submitting your revision, we need you to address these additional requirements.

1. Please ensure that your manuscript meets PLOS ONE's style requirements, including those for file naming. The PLOS ONE style templates can be found at

[https://journals.plos.org/plosone/s/file?id=wjVg/PLOSOne_formatting_sample_main_body.pdf](https://journals.plos.org/plosone/s/file?id=wjVg/PLOSOne_formatting_sample_main_body.pdf) and [https://journals.plos.org/plosone/s/file?id=ba62/PLOSOne_formatting_sample_title_authors_affiliations.pdf](https://journals.plos.org/plosone/s/file?id=ba62/PLOSOne_formatting_sample_title_authors_affiliations.pdf)

**Response 1:** The manuscript meets the journal style requirements.

2. In your Methods section, please provide additional information regarding the permits you obtained for the work. Please ensure you have included the full name of the authority that approved the field site access and, if no permits were required, a brief statement explaining why.

**Response 2:** We don't need permission to work. Because we are the staff of the China Geological Survey, the research area is within our work area, and we have carried out work in accordance with the requirements and reasonable compliance.

3. Thank you for stating the following financial disclosure:

"This research was funded by the Science and Technology Innovation Fund Project of Natural Resources Integrated Survey Command Center (KC20230015, KC20220007), the China Geological Survey Project (DD20230484), the National Natural Science Foundation of China (U2003109) and the Natural Science Foundation of Xinjiang Uygur Autonomous Region (2022D01A149)."

Please state what role the funders took in the study.  If the funders had no role, please state: "The funders had no role in study design, data collection and analysis, decision to publish, or preparation of the manuscript."

If this statement is not correct you must amend it as needed.

Please include this amended Role of Funder statement in your cover letter; we will change the online submission form on your behalf.

**Response 3:** Quan Xu was supported by the Science and Technology Innovation Foundation of the Command Center for Comprehensive Survey of Natural Resources (KC20230015), the National Natural Science Foundation of China (U2003109), and the Natural Science Foundation of Xinjiang Uygur Autonomous Region (2022D01A149). He played a role in data collection, methodology, and writing – original draft. Junling He was supported by the Science and Technology Innovation Fund of the Command Center for Integrated Survey of Natural Resources (KC20220007) and the Geological Survey of China (DD20230484). He played a role in writing – review & editing.

4. In this instance it seems there may be acceptable restrictions in place that prevent the public sharing of your minimal data. However, in line with our goal of ensuring long-term data availability to all interested researchers, PLOS’ Data Policy states that authors cannot be the sole named individuals responsible for ensuring data access ([http://journals.plos.org/plosone/s/data-availability#loc-acceptable-data-sharing-methods](http://journals.plos.org/plosone/s/data-availability#loc-acceptable-data-sharing-methods)).

Data requests to a non-author institutional point of contact, such as a data access or ethics committee, helps guarantee long term stability and availability of data. Providing interested researchers with a durable point of contact ensures data will be accessible even if an author changes email addresses, institutions, or becomes unavailable to answer requests.

Before we proceed with your manuscript, please also provide non-author contact information (phone/email/hyperlink) for a data access committee, ethics committee, or other institutional body to which data requests may be sent. If no institutional body is available to respond to requests for your minimal data, please consider if there any institutional representatives who did not collaborate in the study, and are not listed as authors on the manuscript, who would be able to hold the data and respond to external requests for data access? If so, please provide their contact information (i.e., email address). Please also provide details on how you will ensure persistent or long-term data storage and availability.

**Response 4:** We have uploaded the data to the sharing platform. And the data sharing instructions in the manuscript have been modified. The data are available from the Open Science Framework database. https://osf.io/a9ezj/ DOI 10.17605/OSF.IO/A9EZJ

5. We note that Figure 1 in your submission contain map images which may be copyrighted. All PLOS content is published under the Creative Commons Attribution License (CC BY 4.0), which means that the manuscript, images, and Supporting Information files will be freely available online, and any third party is permitted to access, download, copy, distribute, and use these materials in any way, even commercially, with proper attribution. For these reasons, we cannot publish previously copyrighted maps or satellite images created using proprietary data, such as Google software (Google Maps, Street View, and Earth). For more information, see our copyright guidelines: [http://journals.plos.org/plosone/s/licenses-and-copyright](http://journals.plos.org/plosone/s/licenses-and-copyright).

We require you to either (1) present written permission from the copyright holder to publish these figures specifically under the CC BY 4.0 license, or (2) remove the figures from your submission:

    a. You may seek permission from the original copyright holder of Figure 1 to publish the content specifically under the CC BY 4.0 license.

We recommend that you contact the original copyright holder with the Content Permission Form ([http://journals.plos.org/plosone/s/file?id=7c09/content-permission-form.pdf](http://journals.plos.org/plosone/s/file?id=7c09/content-permission-form.pdf)) and the following text:

“I request permission for the open-access journal PLOS ONE to publish XXX under the Creative Commons Attribution License (CCAL) CC BY 4.0 ([http://creativecommons.org/licenses/by/4.0/](http://creativecommons.org/licenses/by/4.0/)). Please be aware that this license allows unrestricted use and distribution, even commercially, by third parties. Please reply and provide explicit written permission to publish XXX under a CC BY license and complete the attached form.”

Please upload the completed Content Permission Form or other proof of granted permissions as an "Other" file with your submission

In the figure caption of the copyrighted figure, please include the following text: “Reprinted from [ref] under a CC BY license, with permission from [name of publisher], original copyright [original copyright year].”

     b. If you are unable to obtain permission from the original copyright holder to publish these figures under the CC BY 4.0 license or if the copyright holder’s requirements are incompatible with the CC BY 4.0 license, please either i) remove the figure or ii) supply a replacement figure that complies with the CC BY 4.0 license. Please check copyright information on all replacement figures and update the figure caption with source information. If applicable, please specify in the figure caption text when a figure is similar but not identical to the original image and is therefore for illustrative purposes only.

The following resources for replacing copyrighted map figures may be helpful:

USGS National Map Viewer (public domain): [http://viewer.nationalmap.gov/viewer/](http://viewer.nationalmap.gov/viewer/)

The Gateway to Astronaut Photography of Earth (public domain): [http://eol.jsc.nasa.gov/sseop/clickmap/](http://eol.jsc.nasa.gov/sseop/clickmap/)

Maps at the CIA (public domain): [https://www.cia.gov/library/publications/the-world-factbook/index.html](https://www.cia.gov/library/publications/the-world-factbook/index.html) and [https://www.cia.gov/library/publications/cia-maps-publications/index.html](https://www.cia.gov/library/publications/cia-maps-publications/index.html)

NASA Earth Observatory (public domain): [http://earthobservatory.nasa.gov/](http://earthobservatory.nasa.gov/)

Landsat: [http://landsat.visibleearth.nasa.gov/](http://landsat.visibleearth.nasa.gov/)

USGS EROS (Earth Resources Observatory and Science (EROS) Center) (public domain): [http://eros.usgs.gov/#](http://eros.usgs.gov/)

Natural Earth (public domain): [http://www.naturalearthdata.com/](http://www.naturalearthdata.com/)

**Response 5:** We have modified Figure 1.

6. Please include captions for your Supporting Information files at the end of your manuscript, and update any in-text citations to match accordingly. Please see our Supporting Information guidelines for more information: [http://journals.plos.org/plosone/s/supporting-information](http://journals.plos.org/plosone/s/supporting-information).

**Response 6:** Supporting information has been added at the end of the manuscript.

Thanks again to the experts and editor. If you have any questions, please feel free to contact us. We must actively change.
