# Supplementary material for: Refining soil nutrient assessment: Incorporating land use boundaries for precision agriculture
Source: PLoS One. 2024 Sep 9;19(9):e0308423. doi: 10.1371/journal.pone.0308423 (PMC11383206; doi:10.1371/journal.pone.0308423)
Supplement: Attachment — Submitted filename: PlosOne Reviewer Report.docx [file pone.0308423.s004.docx]

**Reviewer Report**

**Title:** Refining Soil Nutrient Assessment: Incorporating Land Use Boundaries for Precision Agriculture

**Authors:** Quan Xu and Junling He

**Journal:** PLOS ONE

**Manuscript ID:** PONE-D-24-18114

**Summary of the Manuscript:**

The manuscript presents a study focused on refining soil nutrient assessment by incorporating land use boundaries for precision agriculture. The study utilizes soil sample data and GIS spatial interpolation analysis in Kashi Prefecture, Xinjiang, to propose a comprehensive evaluation method for assessing soil nutrient levels at the plot level.

**Detailed Review:**

**Introduction**

**Strengths:**

- The introduction effectively establishes the importance of soil nutrient assessment for agricultural productivity and sustainability.
- The authors provide a well-structured literature review, highlighting significant advancements in the field and contextualizing their research within existing studies.
- The objective of the study is clearly stated, focusing on refining soil nutrient assessment by incorporating land use boundaries, which aligns with the goal of precision agriculture.

**Areas for Improvement:**

1. **Inclusion of Recent Studies:**
   - The authors have included more recent studies to provide a broader context and highlight the latest advancements in the field. This enhances the manuscript by demonstrating awareness of current research and positioning the study within the broader academic conversation.
2. **Overview of GIS Techniques:**
   - The manuscript now provides a brief overview of GIS spatial interpolation techniques and explains how plot boundary information was integrated.
3. **Clarification of Technical Terms:**
   - Key technical terms have been defined to ensure accessibility for a broader audience.
4. **Strengthening the Study's Rationale:**
   - The rationale and impact of the study have been strengthened, emphasizing the importance of effective soil nutrient management for optimizing crop yields and sustainable agricultural practices.
5. **Highlighting Novelty and Contributions:**
   - The novelty and contributions of the study have been highlighted, focusing on the integration of plot boundary information into the spatial interpolation of soil nutrient levels.

**Methodology**

**Strengths:**

- The methodology is well-detailed, describing the study area, soil sampling and analysis, spatial interpolation methods, and the comprehensive evaluation method.
- The use of GIS technology to integrate soil nutrient data with spatial interpolation results enhances the precision of soil nutrient evaluation.

**Areas for Improvement:**

1. **Details on Spatial Interpolation Techniques:**
   - More specific details on the spatial interpolation techniques and parameters used have been provided, ensuring reproducibility.
2. **Validation of Spatial Interpolation Methods:**
   - The manuscript now suggests including a section on the validation of spatial interpolation methods, demonstrating accuracy and reliability.
3. **Practical Applications:**
   - The practical applications of the findings have been expanded, including specific recommendations for farmers and agricultural managers.

**Results**

**Strengths:**

- The results section presents a clear and comprehensive analysis of soil nutrient levels and their spatial distribution.
- The descriptive statistical analysis provides an understanding of overall soil nutrient levels and their variation within the study area.
- Spatial distribution maps and analysis effectively illustrate nutrient distribution patterns and highlight areas with different nutrient levels.
- The comprehensive evaluation categorizes soil nutrient levels into different grades, offering practical implications for soil management and fertilization practices.

**Areas for Improvement:**

1. **Clarity of Figures and Maps:**
   - Figures and maps are now clear and well-labeled, enhancing readability and understanding of spatial distribution patterns.
2. **Explanations of Spatial Distribution Patterns:**
   - The manuscript provides more detailed explanations of spatial distribution patterns and their implications for soil management and crop production.

**Discussion**

**Strengths:**

- The discussion emphasizes the practical applications of the findings for precision agriculture.
- It provides a clear summary of the study's contributions and the importance of incorporating plot boundary information into soil nutrient assessments.

**Areas for Improvement:**

1. **Objective Weighting Methods:**
   - The manuscript discusses the potential use of objective weighting methods for assigning values to soil nutrient indicators in future research.
2. **Validation of Spatial Interpolation Results:**
   - A section on the validation of spatial interpolation results using training and validation sets has been suggested to improve accuracy.

**Conclusion**

**Strengths:**

- The conclusion effectively summarizes the key findings of the study and provides recommendations for future research and practical applications.
- It highlights the importance of refining and enhancing the comprehensive evaluation method for broader applicability and accuracy.

**Areas for Improvement:**

1. **Future Research Directions:**
   - Specific areas for future research have been suggested to further refine and enhance the comprehensive evaluation method.

**Presentation and Language**

**Strengths:**

- The manuscript is presented in an intelligible fashion and written in standard English.
- It is well-organized, with a clear structure guiding the reader through the research process and findings.
- Technical terms are used accurately and consistently throughout the manuscript.

**Recommendations for Improvement:**

1. **Proofreading:**
   - A thorough proofreading has been conducted to catch any minor grammatical or typographical errors.
2. **Simplifying Complex Sentences:**
   - Overly complex sentences have been simplified to improve readability.
3. **Visual Aids:**
   - Figures, tables, and maps are clearly labeled and include descriptive captions.

**Data Availability**

**Strengths:**

- The manuscript includes a data availability statement specifying where and how the raw data and GIS files can be accessed.

**Final Recommendation**

Based on the detailed review, I find that the manuscript presents significant contributions to the field of soil nutrient assessment and precision agriculture. The methodology is robust, and the results offer valuable insights for improving soil management and crop production. The authors have addressed all of the comments provided in the review report, enhancing the manuscript's clarity, reliability, and practical relevance.

**Recommendation:** Accept as it is.

**Sincerely,**

**Bright Fafali Dogbey**
